# Modelling image quality over extended [^18^F]FDG uptake times for low-dose positron emission mammography

**DOI:** 10.3389/fonc.2026.1880803

**Published:** 2026-09-14

**Authors:** Elliott De Benetti, Tobias Preckel, Apichart Linhananta, Brandon Baldassi, Borys Komarov, Harutyun Poladyan, Vivianne Freitas, Alla Reznik, Oleksandr Bubon

**Affiliations:** 1 Department of Physics, Lakehead University, Thunder Bay, ON, Canada; 2 Department of Medical Engineering, Pforzheim University, Pforzheim, Germany; 3 Radialis Inc., Thunder Bay, ON, Canada; 4 Temerty Faculty of Medicine, Joint Department of Medical Imaging, University of Toronto, Toronto, ON, Canada; 5 Thunder Bay Regional Health Research Institute, Thunder Bay, ON, Canada; 6 Ontario Institute for Cancer Research, Toronto, ON, Canada

**Keywords:** [^18^F]FDG PEM, breast cancer, breast imaging, delayed imaging, image quality, kinetic modelling, positron emission mammography

## Abstract

**Introduction:**

This study investigated the effect of [^18^F]fluorodeoxyglucose ([^18^F]FDG) uptake time on image quality in low-dose positron emission mammography (PEM) using kinetic modelling.

**Methods:**

Fifteen malignant lesions from thirteen women newly diagnosed with breast cancer were imaged using low-dose [^18^F]FDG PEM. Tumour and normal breast tissue time-activity curves (TACs) were generated using a population-based arterial input function (AIF) and a two-tissue compartmental model. Modelling was performed at [^18^F]FDG doses of 1, 2, 5, and 10 mCi (37, 74, 185, and 370 MBq), and image quality was assessed using contrast-to-noise ratio (CNR) over a 6-hour uptake period.

**Results:**

Extending uptake times beyond the 1-hour clinical standard significantly improved CNR across all doses, with comparable proportional gains. Relative to 1 hour post-injection, CNR increased by approximately 30% at 1.5 hours, 53% at 2 hours, and 77% at 3 hours, although uncertainty widened at 3 hours and beyond, particularly at the lowest dose (1 mCi). Relatively small variations in uptake time were also found to produce substantial changes in the standardized uptake value (SUV).

**Discussion:**

These findings demonstrate that extending uptake times by even 30 minutes beyond the 1-hour clinical standard may significantly enhance image CNR for low-dose [^18^F]FDG PEM. The sensitivity of SUV to small variations in uptake time supports the need to closely match uptake time between baseline and follow-up scans in treatment response assessment. Overall, these modelling results encourage further clinical research into optimizing uptake time protocols, which may help support the broader adoption of low-dose PEM in future work.

**Clinical Trials Registration:**

https://clinicaltrials.gov/study/NCT03520218, identifier NCT03520218.

## 1 Introduction

There is a growing clinical need to reduce the radiation dose associated with [^18^F]FDG PET (Positron Emission Tomography) imaging while improving diagnostic performance. Recent innovations in detector technologies and scanner geometries, such as total-body and organ-specific PET, address this need by increasing detector solid-angle coverage to improve signal collection efficiency (1–3). Total-body PET achieves this by extending the axial field of view (FOV) to encompass the entire body, whereas organ-specific PET tailors the FOV to the target organ (e.g., breast or brain). In both cases, the resulting increase in geometric efficiency enhances the detection rate of coincident photon pairs, improving the signal-to-noise ratio and image contrast (1–3)—thereby enabling either lower injected activities or shorter acquisitions (or both) without compromising lesion detectability (4).

Positron emission mammography (PEM) is particularly relevant to an unmet clinical need in breast cancer diagnosis among women with dense breast tissue. In this population, conventional X-ray mammography often has reduced sensitivity because of the low intrinsic X-ray attenuation contrast between fibroglandular tissue and tumours, resulting in a “masking effect” whereby dense breast tissue obscures malignant lesions (5, 6). This masking effect yields a high number of both false-negative and false-positive mammography findings, which can lead to late cancer diagnosis, patient anxiety, and unnecessary follow-up procedures (7). As a functional imaging modality, [^18^F]FDG PEM is not subject to this density-dependent attenuation masking, since it visualizes the metabolic activity of breast tissue rather than its anatomical structure. Consistent with this, PEM has demonstrated the ability to detect small lesions obscured on digital X-ray mammography and to decrease false-positive MRI findings (8, 9).

Breast density is, in addition, an independent risk factor for cancer development, creating a paradox in which the women who face the greatest oncological risk are also those for whom X-ray mammography screening is least sensitive (10). This makes supplemental or alternative screening in dense breasts an attractive application for PEM, and its feasibility in women at elevated risk has been supported by early work (11). For example, a preliminary screening study of 265 women using PEM and [^18^F]FDG doses of 141–304 MBq (3.8–8.2 mCi) showed a cancer detection rate of 2.3% compared to 0.31% using a combination of mammography and physical examination (12). However, since screening requires routine repeated imaging of asymptomatic women, the acceptable radiation burden is correspondingly lower. Any role for PEM in this setting is therefore contingent on achieving an effective dose comparable to established breast screening modalities, including full-field digital mammography (FFDM), digital breast tomosynthesis (DBT) and combined FFDM-DBT.

For [^18^F]FDG, the effective dose is approximately 0.019 mSv/MBq (13). Therefore, injected activities of 1 mCi (37 MBq) and 2 mCi (74 MBq) yield effective doses of roughly 0.7 mSv and 1.4 mSv, respectively. By comparison, FFDM has an effective dose of about 0.5 mSv, while FFDM-DBT has an effective dose of about 1.2 mSv (14). Although a slightly elevated radiation dose in medical imaging procedures may be acceptable when clinical benefits outweigh the risk, optimizing PEM imaging for low doses (i.e., ≤ 2 mCi) remains necessary for screening. More specifically, a dose of 1 mCi is ideal—equivalent to a tenfold reduction relative to standard whole-body (WB) PET—as it would keep the effective dose within a range considered acceptable for screening and supplemental imaging in women with dense breasts.

One proposed approach for improving low-dose image quality in high-sensitivity systems is delayed imaging, in which scans are acquired beyond the standard 1-hour [^18^F]FDG uptake time to allow more time for differential tracer uptake (15). The rationale is promoting [^18^F]FDG washout from normal tissue (reducing background activity) and, in some cases, further increasing tumour activity—therefore improving tumour-to-background uptake ratio (16, 17). Delayed [^18^F]FDG PET has been shown to enhance visualization of otherwise less conspicuous lesions in anatomical regions with intrinsically high background activity, where small differences in uptake are easily obscured (18). This is also particularly relevant in the breast (19–22), where FDG-avid benign conditions such as inflammation, infection, or fibroadenomas can lead to false-positive findings, and where false negatives may arise in cancer subtypes with lower FDG uptake (e.g., lobular carcinomas) or when elevated background activity in dense fibroglandular tissue obscures small tumours (23).

Conversely, delayed imaging comes at the expense of lower count rates and longer acquisition times as ^18^F decays. Given this trade-off of improved target-to-background ratios and decreased count rates, optimizing uptake time in low-dose imaging is crucial for maximizing image quality, particularly the contrast-to-noise ratio (CNR) (24). CNR, a key quantitative metric for evaluating image quality in PET studies, describes the visibility of a tumour relative to background noise, where low CNR can adversely affect diagnostic interpretation and confidence (25). Low CNR values have also been shown to significantly impact the accuracy of SUV measurements, a standardized measure of tissue metabolic activity in clinical practice (26, 27). Thus, in low-dose or delayed PET imaging, when radiotracer activity is reduced, maximizing CNR becomes critical to preserve the reliability of visual interpretation and SUV measurements in reconstructed PET images.

To date, no studies have evaluated delayed breast tumour imaging with new-generation organ-targeted PET systems at low [^18^F]FDG doses (i.e., ≤2 mCi). Using images acquired on a Radialis PET Imager, an organ-targeted PET system configurable for PEM (9), we performed a modelling study to assess image quality over a 6-hour uptake window for low-dose [^18^F]FDG PEM, quantifying changes in CNR by fitting kinetic models to clinical image data. Optimal uptake times were identified across varying [^18^F]FDG doses by extending this framework to simulate different injected doses and compare the modelled CNR.

## 2 Materials and methods

### 2.1 Imaging system

The Radialis PET Imager, configured for breast imaging, is shown in **Figure 1**. This system is a compact, mobile PET imager, featuring two large-area (174 × 232 mm^2^) high-resolution planar gamma detectors (referred to as “detector heads”) mounted on an adjustable motorized gantry with rotational capability (28). The detector heads are positioned on either side of the immobilized (i.e., not compressed) breast, providing full coverage during image acquisition in both craniocaudal (CC) and mediolateral oblique (MLO) orientations.

**Figure 1:**
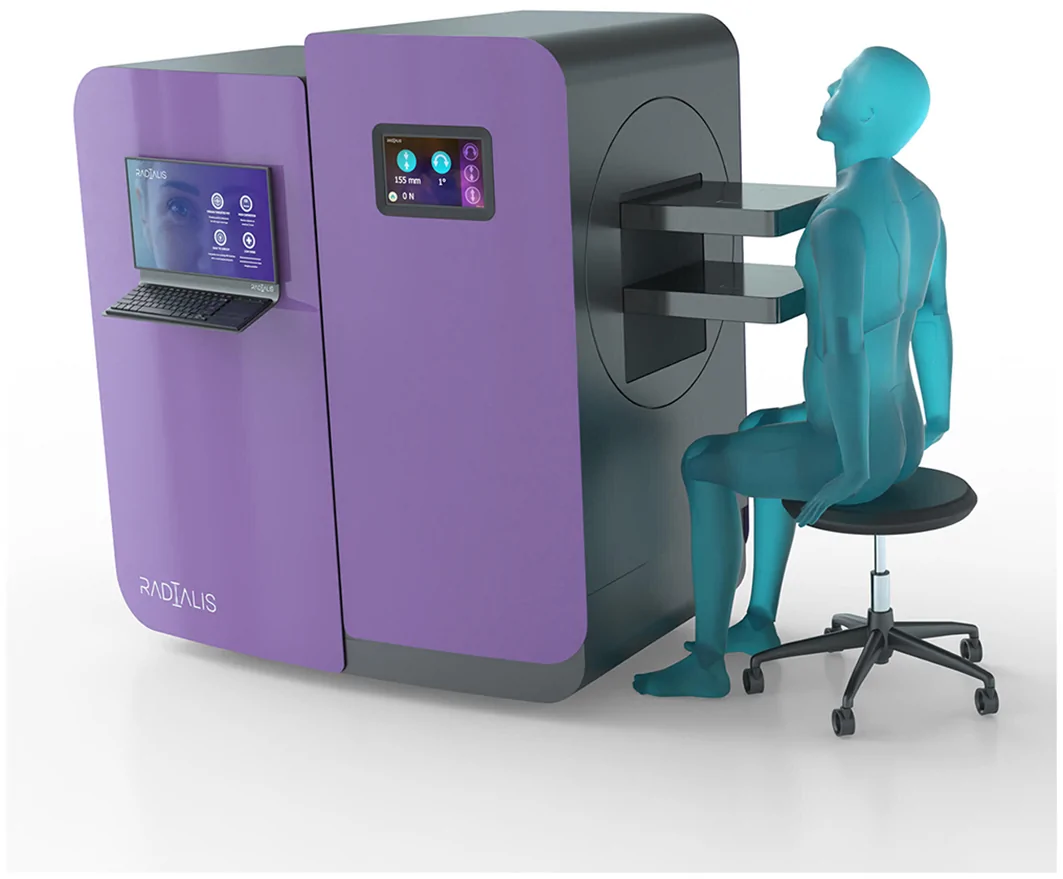
The Radialis PET Imager configured for breast imaging.

### 2.2 Participant data

Modelling data were drawn from a pilot, single-centre clinical evaluation performed at the University Health Network Princess Margaret Cancer Centre (9), registered on ClinicalTrials.gov (NCT03520218). Between January 2020 and August 2022, 36 participants with histopathology-confirmed breast cancer were enrolled in the pilot evaluation and received an [^18^F]FDG dose of 1, 2, or 5 mCi to assess the performance of the Radialis PEM system under reduced-dose conditions.

For the present modelling work, we included only participants who underwent dual-time-point breast acquisitions (two Radialis PEM examinations on the same day at different post-injection times), thereby meeting the modelling requirements. The final modelling dataset included images from 13 participants with 15 malignant breast lesions, as two participants had bilateral lesions. **Table 1** outlines the general parameters of the thirteen participants included in the study. The 13 included participants received either a 2 mCi (n = 7) or 5 mCi (n = 6) [^18^F]FDG dose.

**Table 1:** General participant information (*n* = 13).

Parameter	Mean	Range
Age (y)	57.8	32.0–85.0
Weight (kg)	76.0	45.4–126.0
Height (m)	1.60	1.49–1.75
Injected dose (mCi)	3.4	1.8–5.3

Cancer types included three lesions of ductal carcinoma *in situ* (DCIS), eleven lesions of invasive ductal carcinoma (IDC), and one lesion of invasive lobular carcinoma (ILC). Among the invasive lesions, histological grade was I/II in four lesions, III in four lesions, and unavailable for the remaining four. Of the three *in situ* lesions, one was classified as nuclear grade II/III and the other two as grade III. Regarding receptor status, eight lesions were estrogen receptor-positive, seven were progesterone receptor-positive, one was triple-negative, and molecular subtype information was unavailable for four lesions.

### 2.3 Acquisition protocol

Imaging was performed in two sessions, approximately 2 and 4 hours post-injection to capture the delayed pharmacokinetics of [^18^F]FDG in breast tissue. Each session included two static acquisitions of 5–7 minutes per view using standard CC and MLO mammographic orientations. For participants with unilateral disease, four images of the affected breast were acquired in the two sessions (a CC and MLO image at each session). For bilateral lesions, four images per breast were obtained, following the same protocol.

Image reconstruction was performed using a 15-iteration Maximum-Likelihood Expectation Maximization (MLEM) algorithm written in C++ that incorporated attenuation, scatter, and solid-angle corrections (29). A 0.8 mm FWHM Gaussian filter was applied in the XY plane at the end of the reconstruction pipeline to reduce image noise. The reconstructed voxels have in-plane voxel dimensions of 0.4 × 0.4 mm, with the axial dimension determined by the detector separation (breast compression distance) divided into 24 equal slices, resulting in axial voxel dimensions ranging from 2.3 to 4.5 mm across all participant images.

### 2.4 Image analysis

All PEM images were visually reviewed in consensus by two breast radiologists trained in nuclear medicine to confirm the visual presence of malignancy prior to quantitative analysis. Quantitative measurements were performed using OsiriX version 14.1 (Pixmeo SARL, Bernex, Switzerland).

#### 2.4.1 Image sampling

Tumour regions of interest (ROIs) were defined using the SUV_peak_ method, where a 1.2-cm-diameter spherical ROI is positioned to maximize mean activity concentration with each lesion (30). All analysed lesions had diameters ≥ 1.2 cm, limiting the inclusion of background tissue within the tumour sampling ROI. Background activity was estimated using three circular ROIs, each approximately 1 cm in diameter (31), positioned within the ipsilateral breast tissue and on the same axial slice as the tumour ROI. Background ROIs were manually placed in regions of uniform uptake and at consistent anatomical locations across images to ensure reproducibility. The mean background activity concentration for each image was computed as the average of the three background ROI means, and the background noise was quantified as the root-mean-square of their standard deviations.

#### 2.4.2 CNR calculation

Image quality was assessed using the contrast-to-noise ratio (CNR), defined as shown in Equation 1:

(1) $$CNR=\frac{\left|T-B\right|}{SD_{B}}$$

where *T* is the mean tumour activity concentration, *B* is the mean background activity concentration, and *SD_B_* is the standard deviation of the background ROIs. Image CNR was calculated separately in each four-image series.

### 2.5 Modelled time-activity curves

Time-activity curves (TACs), which describe the change in [^18^F]FDG over time in a given tissue, were modelled for tumour and normal breast tissue over an extended 6-hour uptake period using a reversible two-tissue compartmental model (32). While an irreversible compartment model commonly employed for TAC modelling up to 1 to 2 hours post-injection would favourably reduce the dimensionality of the parameter space, we opted for the reversible model in this study as the added dephosphorylation parameter (*k*_4_, defined below) has been shown to have a greater impact on uptake curves at later time points (20). All TAC generation was performed using NumPy and SciPy in Python 3.12.

#### 2.5.1 Two-tissue compartment model

The model describes [^18^F]FDG exchange between arterial plasma (*C_P_*), free (unphosphorylated) tissue (*C*_1_), and phosphorylated (trapped) tissue compartments (*C*_2_). The summed [^18^F]FDG concentration in the compartments then represent the [^18^F]FDG concentration quantified within a given region of a PET (in this case, PEM) image (*C_PET_*). The rate constants *K*_1_ and *k*_2_ relate to the transport of [^18^F]FDG from plasma compartment into and out of tissue, while *k*_3_ and *k*_4_ describe phosphorylation ([^18^F]FDG trapping) and dephosphorylation ([^18^F]FDG release), respectively. A vascular volume fraction (*V_B_*) of 0.04 was assigned to breast tissue based on published values (33). A visual representation of the compartment model is shown in **Figure 2**. Tumour and normal breast tissue were each represented as a single homogeneous compartment, consistent with standard FDG kinetic-modelling practice, to capture the dominant washout behaviour that governs the background term in the CNR.

**Figure 2:**
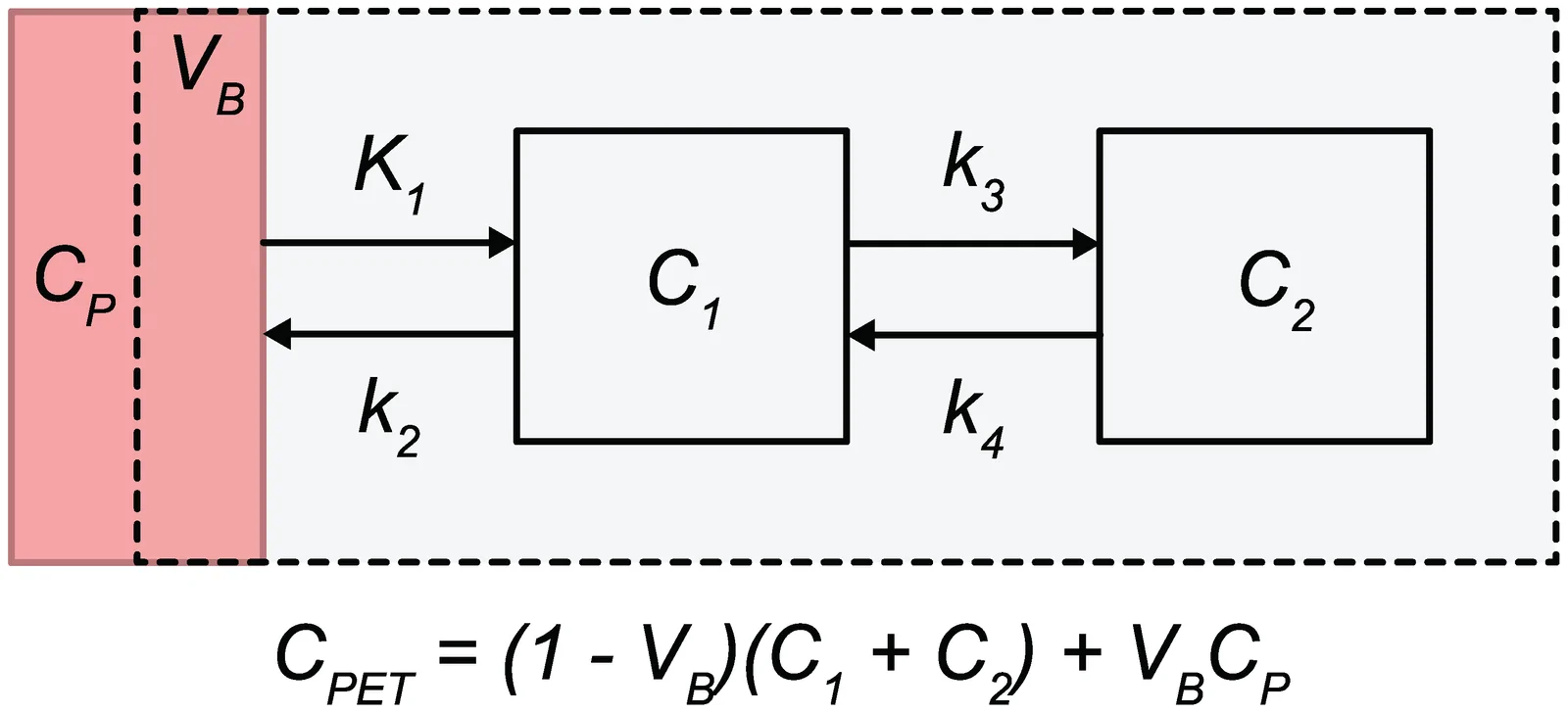
The two-tissue compartment model describing the pharmacokinetics of [^18^F]FDG. Compartment *C_P_* denotes the [^18^F]FDG in the arterial plasma, while *C*_1_ and *C*_2_ denote free and trapped [18F]FDG in the tissue. The vascular volume fraction, *V_B_*, is the fractional contribution of tissue vasculature to the total [^18^F]FDG concentration of the tissue. Rate constants *K*_1_ (mL·g^-1^·min^-1^) and *k*_2_ (min^-1^) correspond to the tissue influx and efflux of [^18^F]FDG, respectively. Rate constants *k*_3_ (min^-1^) and *k*_4_ (min^-1^) correspond to the phosphorylation (trapping) and dephosphorylation of [^18^F]FDG, respectively. *C_PET_* denotes the combined [^18^F]FDG concentration measurable within a given tissue region of a PET image.

#### 2.5.2 Arterial input function

Since no blood samples were collected during the clinical evaluation, and no major arteries were visible in the FOV, the plasma [^18^F]FDG input, *C_P_*, was represented using a population-based AIF (34). The population-based AIF was scaled to each participant using the approximate initial plasma concentration, $C_{P}^{*}\left(0\right)$, calculated as described by Naganawa et al. (34):

(2) $$C_{P}^{*}\left(0\right)=\frac{injected\;dose\left[MBq\right]}{1.18\cdot {\left(height\left[m\right]\right)}^{0.68}{\left(weight\left[kg\right]\right)}^{0.45}}$$

After scaling the AIF to each participant, it was then convolved with a 1-minute-long rectangular function to account for the [^18^F]FDG injection duration. To prevent convolution boundary artifacts, the AIF and rectangular function were first edge-padded and then cropped post-convolution to the final 0–6-hour interval.

### 2.6 Modelled CNR curves

To model CNR over the extended uptake period, a time-dependent estimate of background noise (*SD_B_*) was required. Since image noise is heavily tied to mean activity concentration, we constructed an empirical noise-activity reference curve specific to the Radialis PEM Imager (**Figure 3**). A 500 mL IV bag uniformly filled with 0.189 mCi (7 MBq) of ^18^F solution was centred between the detectors (at 118 mm separation), and imaged with 7-minute acquisitions taken hourly for 16 hours. In this case the IV bag was used as a homogeneous calibration object, serving the same practical role as a conventional uniform phantom. Each image was reconstructed using the same protocol as the clinical breast images. For every acquisition, a rectangular ROI was drawn on the central slice of the IV bag to measure the mean background activity concentration and corresponding standard deviation. Using least squares regression, the linear function in Equation 3.

**Figure 3:**
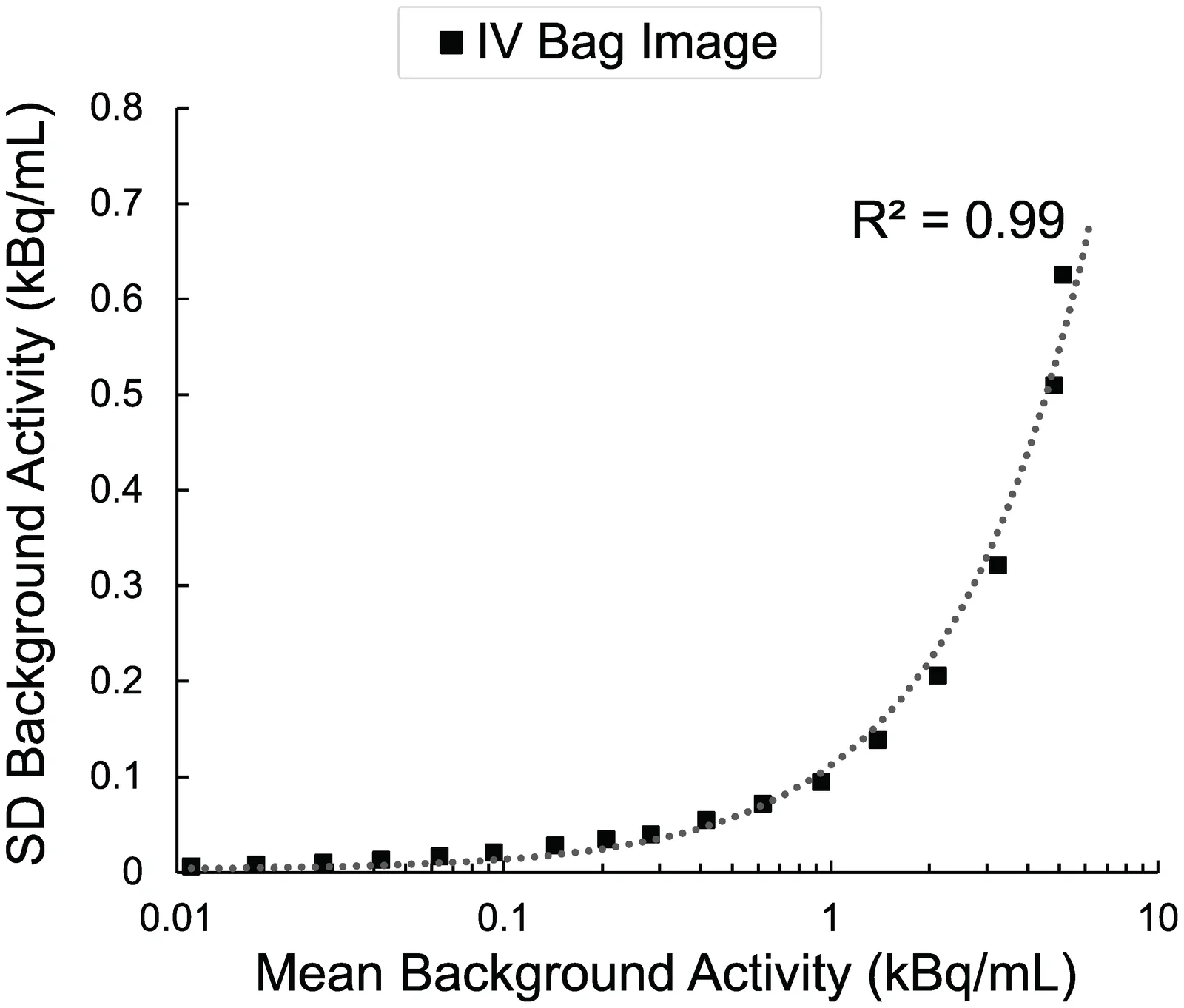
Experimentally derived noise-activity reference curve specific to the Radialis PET Imager configured for PEM. Each point corresponds to the sampled standard deviation (SD) and mean background activity concentration in the image of a uniformly filled IV bag as it decayed.

(3) σ(μ)=0.109·μ

was fit (R^2^ = 0.99), where *μ* [kBq/mL] is the mean background activity concentration measured in the IV bag ROI and σ(*μ*) [kBq/mL] is the corresponding predicted standard deviation.

For each participant image series, rather than applying the reference curve directly, we added a participant-specific adjustment term (Equation 4),

(4) $$\sigma_{a}\left(\mu \right)=0.109\cdot \mu +a$$

where *a* is the adjustment term that is optimized using least squares regression to maximize the fit with the measured standard deviation of background activity for each series. This ensured that the estimated *SD_B_* reflected any baseline noise or inhomogeneity of unique to the image series while preserving the linear relationship determined in the calibration reference curve experiment.

The adjusted noise curve was then used with the background breast TAC, *B*(*t*) as an input to enable the indirect estimation of background noise as a function of uptake time (Equation 5):

(5) $$SD_{B}\left(t\right)\approx \sigma_{a}\left(B\left(t\right)\right).$$

Thus, CNR curves were computed as (Equation 6):

(6) $$CNR\left(t\right)=\frac{\left|T\left(t\right)-B\left(t\right)\right|}{\sigma_{a}\left(B\left(t\right)\right)},$$

where *T*(*t*) and *B*(*t*) are the modelled tumour and background activity concentrations, respectively. This procedure generated a continuous CNR profile for each set of tumour and background TACs.

### 2.7 Kinetic parameter optimization

In contrast to conventional kinetic analyses that use full dynamic acquisitions (20), our modelling relied on four delayed static PEM images per lesion (two views each at around 2 and 4-hours post-injection) to model each TAC and CNR curve. This limited sampling necessitated a carefully constrained optimization strategy to recover plausible tumour and background kinetics. To jointly estimate the kinetic behaviour of tumour and normal (background) breast tissue, we optimized an eight-parameter vector (Equation 7):

(7) $$\theta =\left[K_{1T},k_{2T},k_{3T},k_{4T},K_{1B},k_{2B},k_{3B},k_{4B}\right]$$

where the subscripts *T* and *B* denote the parameters belonging to the tumour and background tissue compartment models, respectively. This vector was optimized using the Sequential Least Squares Programming (SLSQP) algorithm included in SciPy (v1.16.3), selected for its ability to handle bounded, nonlinear objective functions consistent with the physiological parameter bounds described below. Each optimization run was subject to a convergence tolerance of 1 × 10^-6^ (SciPy default), defined as the minimum change in the objective function between iterations. To mitigate convergence to local minima, SLSQP was applied within a multi-start framework, covered in section 2.8.

The optimization objective function that SLSQP minimized was defined as:

(8) $$J\left(\theta \right)=\frac{100\%}{4}\sum_{i=1}^{4}\left(\frac{1}{3}\left|\frac{T_{i,image}-T_{i,model\left(\theta \right)}}{T_{i,image}}\right|+\frac{1}{3}\left|\frac{B_{i,image}-B_{i,model\left(\theta \right)}}{B_{i,image}}\right|+\frac{1}{3}\left|\frac{CNR_{i,image}-CNR_{i,model\left(\theta \right)}}{CNR_{i,image}}\right|\right),$$

where for a given parameter set *θ*; *T*, *B*, and *CNR* denote tumour activity, background activity, and contrast-to-noise ratio, respectively; subscripts *image* and *model*(θ) denote measured imaging and modelled values for a given parameter set; and *i* indexes the four imaging time points. Equal weighting of the three terms (tumour activity, background activity, and CNR) ensures balanced fitting across the different measurements. We acknowledge that including the CNR term influences the recovered parameters, favouring fidelity to the measured CNR over marginally closer fits to the individual activity curves; as the modelled CNR is the primary quantity of interest, this trade-off was considered appropriate.

Since only four imaging time points were available, physiologically plausible bounds were imposed on all kinetic parameters to guide optimization. These bounds (listed in **Table 2**) specified the minimum and maximum allowed individual parameter values, which the optimizer could estimate. Parameter ranges for normal breast tissue were derived from a dataset containing 49 kinetic parameter sets (from 23 participants) reported by Wangerin et al. (20). Breast tumour parameter bounds were derived from the Wangerin et al. dataset, along with one other study that published summary statistics of breast tumour kinetic parameters (20, 35). A flowchart summarizing the parameter optimization pipeline is shown in **Figure 4**.

**Table 2:** Kinetic model parameter optimization bounds and the guiding references.

Parameter	Lower bound	Upper bound	Ref.
*K*_1_*_T_* (mL·g^-1^·min^-1^)	0.005	0.742	(20, 35)
*k*_2_*_T_* (min^-1^)	0.032	2.916	(20, 35)
*k*_3_*_T_* (min^-1^)	0.0001	0.164	(20, 35)
*k*_4_*_T_* (min^-1^)	0.001	0.010	(20)
*K*_1_*_B_* (mL·g^-1^·min^-1^)	0.001	0.202	(20)
*k*_2_*_B_* (min^-1^)	0.008	0.902	(20)
*k*_3_*_B_* (min^-1^)	0.0001	0.263	(20)
*k*_4_*_B_* (min^-1^)	0.001	0.010	(20)

Subscripts *T* and *B* denote the parameters belonging to the tumour and background tissue compartment models, respectively.

**Figure 4:**
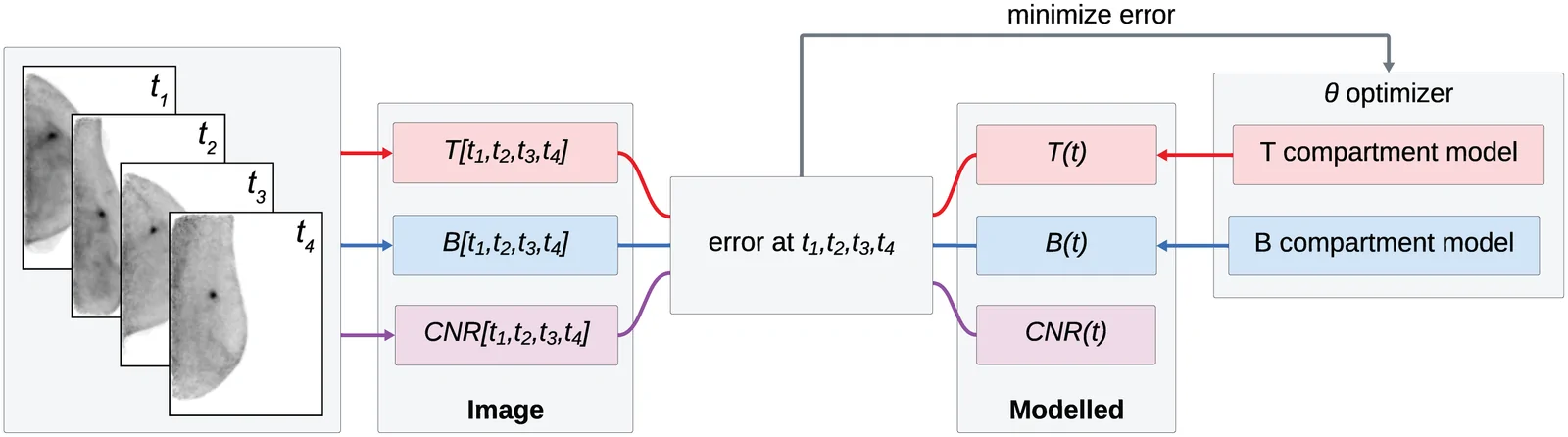
Flowchart of the kinetic parameter (*θ*) optimization pipeline. Tumour and background breast activity concentration is denoted by the letters ‘*T*’ and ‘*B*’, respectively. The post-injection time points of the four-image-series are denoted as *t*_1_, *t*_2_, *t*_3_, and *t*_4_. Error is defined as the mean absolute percentage error across tumour activity, background activity, and CNR, for each of the four time points as defined in Equation 8. Modelled CNR denoted *CNR*(*t*) was calculated using *T*(*t*) and *B*(*t*) as defined in Equation 6.

### 2.8 Kinetic parameter exploration

With only four delayed imaging time points available for each four-parameter TAC, a single optimized parameter set could not fully characterize the underlying tumour and background kinetics. To capture a range of additional physiologically plausible solutions, multiple parameter sets were generated for each image set by exploring a bounded parameter space. Thus, it was assumed that, through the use of physiologically constrained optimizer bounds and multiple plausible parameter sets, the resulting TAC and CNR curve data provided a sufficiently robust basis for subsequent analysis despite the parameter estimates originating from an underdetermined system.

First, an optimal baseline parameter estimate, *θ*_0_, was obtained using a multi-start optimization strategy. A total of 128 initial parameter vectors were generated using Latin Hypercube Sampling to uniformly cover the eight-dimensional search space. Each vector was optimized using the constrained SLSQP routine described in section 2.7, and the solution yielding the lowest objective function value was designated as the baseline optimum (*θ*_0_). Using this baseline, we then defined the criterion for an acceptable alternative parameter vector, where a candidate *θ* was considered acceptable if its objective value *J*(*θ*) fell within an additional 5% mean absolute percentage error of the baseline *J*(*θ*_0_) (Equation 9):

(9) $$J\left(\theta \right)\leq J\left(\theta_{0}\right)+5\%.$$

This threshold of an additional 5% mean absolute percentage error was chosen empirically to capture visually plausible curves describing the observed data, rather than to represent formal statistical confidence bounds. Given the limited number of samples to fit each parameter set, rigorous confidence intervals were not estimable; instead, we present this exploratory range to illustrate parameter sensitivity and the envelope of the extremes of plausible model behaviours.

With this acceptance criterion established, we searched for the boundary of the plausible parameter space along each of the eight kinetic parameters in *θ*, performing a separate min/max search in each direction (toward the parameter’s minimum and maximum physiological bounds). In this iterative procedure, the target parameter was incrementally shifted toward the bound in question while the remaining seven parameters were re-optimized at each step using diverse initial guesses (local perturbations, Latin Hypercube Sampling, and alternative solutions from the *θ*_0_ multi-start optimization). This shift-and-reoptimize cycle continued until the acceptance criterion was no longer satisfied or the physiological bound was reached, at which point the last acceptable value was recorded as *θ_i_*,*_min_*, or *θ_i_*,*_max_*. This process, repeated for all eight parameters in both directions, yielded 16 boundary parameter vectors, which together with the baseline optimum *θ*_0_ produced 17 total parameter vectors per image set, collectively representing a broad range of kinetic behaviours consistent with the imaging data and physiologic bounds.

### 2.9 Alternate dose simulations

Since the kinetic parameters of the two-tissue compartmental model for [^18^F]FDG reflect the rate constants of underlying transport and metabolism, they were assumed not to vary over the injected-dose range considered here. Accordingly, only the AIF needed to be updated to simulate alternative doses. This was implemented by adjusting the injected dose term in the numerator of the population-based AIF function (Equation 2), $C_{P}^{*}\left(0\right)$. In addition to the actual doses (2 mCi or 5 mCi) used in the clinical study, three additional doses were simulated for each participant, yielding TACs and CNR curves for 1, 2, 5, and 10 mCi.

### 2.10 Assessment of detectability

The theoretical confidence of tumour detectability was assessed using CNR under the Rose Criterion, which states that a CNR must exceed 3–5 for confident detection (24, 36). Despite the criterion’s limitations in medical imaging (based on the detection of circular objects against uniform backgrounds with non-structured noise), it remains a useful indicator for assessing confidence in tumour detection. Detectability confidence under the Rose criterion was evaluated both for the baseline solution and across the full envelope, with a lesion considered confidently detectable at a given dose and time only if all 17 parameter vectors yielded CNR ≥ 5.

### 2.11 Statistical analysis

All statistical analyses were performed in R (v4.6.1; R Core Team 2026) using the lme4, lmerTest, and emmeans packages. The unit of analysis was each breast lesion (n = 15), drawn from the 13 participants, two of whom contributed bilateral lesions. Inferential testing was restricted to the baseline parameter solution (*θ*_0_), yielding one CNR curve per lesion per simulated dose. The 16 boundary parameter sets (*θ_min/max_*) previously described in section 2.8 were not treated as independent observations, as they constitute a deterministic parameter-uncertainty envelope rather than independent replicates and therefore were used only for descriptive sensitivity analysis. Analyses were conducted on the natural-log scale, since the effect of interest was the relative (percentage) change in CNR from the 1-hour reference; on the log scale such multiplicative effects become additive and are therefore appropriately modelled by a linear model. This was supported by the observation that within-lesion differences from the 1-hour reference were right-skewed on the raw scale but showed no evidence against normality as log ratios (in Shapiro–Wilk tests and visual inspection of quantile–quantile plots).

CNR at the standard 1-hour uptake time was compared with CNR at three delayed uptake times (1.5, 2, and 3 hours) using a linear mixed-effects model of log CNR, with time point and dose as fixed effects and random intercepts for lesions nested within participants. This structure accounts for the repeated measurement of each lesion across time points and simulated doses, and for the non-independence of bilateral lesions from the same participant. Random slopes were not estimable with the limited sample size of 15 lesions. An uptake time-by-dose interaction was fitted first to test whether the proportional change in CNR from the 1-hour reference differed across the four simulated doses; this interaction was not significant (F(9, 210) = 0.19, *p* = 0.995). The primary model thus estimated the uptake time effect pooled across doses. Pre-planned contrasts compared each delayed uptake time point with the 1-hour reference, with Dunnett adjustment for multiplicity. Estimates were back-transformed from the log scale and are reported as percent change with 95% confidence intervals. Two-sided *p*-values below 0.05 were considered statistically significant.

Two robustness analyses were pre-specified. First, all uptake time comparisons were repeated using paired Wilcoxon signed-rank tests on the within-lesion log differences, Holm-adjusted within dose group, to confirm that conclusions did not depend on parametric assumptions made in the linear mixed model. Second, the model was refit using each lesion’s median across all 17 parameter vectors to confirm that conclusions were stable across the parameter-uncertainty envelope. Peak CNR and peak timing were excluded from inferential models and are reported descriptively to avoid conflating the effect of delayed imaging with the effect of taking a maximum. For a detailed description of the statistical analysis, please refer to the **Supplementary Material**.

## 3 Results

### 3.1 Modelled time-activity curves

Tumour TACs had significantly greater magnitude than the normal background tissue TACs due to higher uptake (**Figure 5**). Tumour activity rises to a peak and then declines, in some cases approaching a shallow semi-plateau over the 6-hour uptake period. Background breast activity follows a different pattern, with a largely monotonic decline that later approaches a low-amplitude plateau.

**Figure 5:**
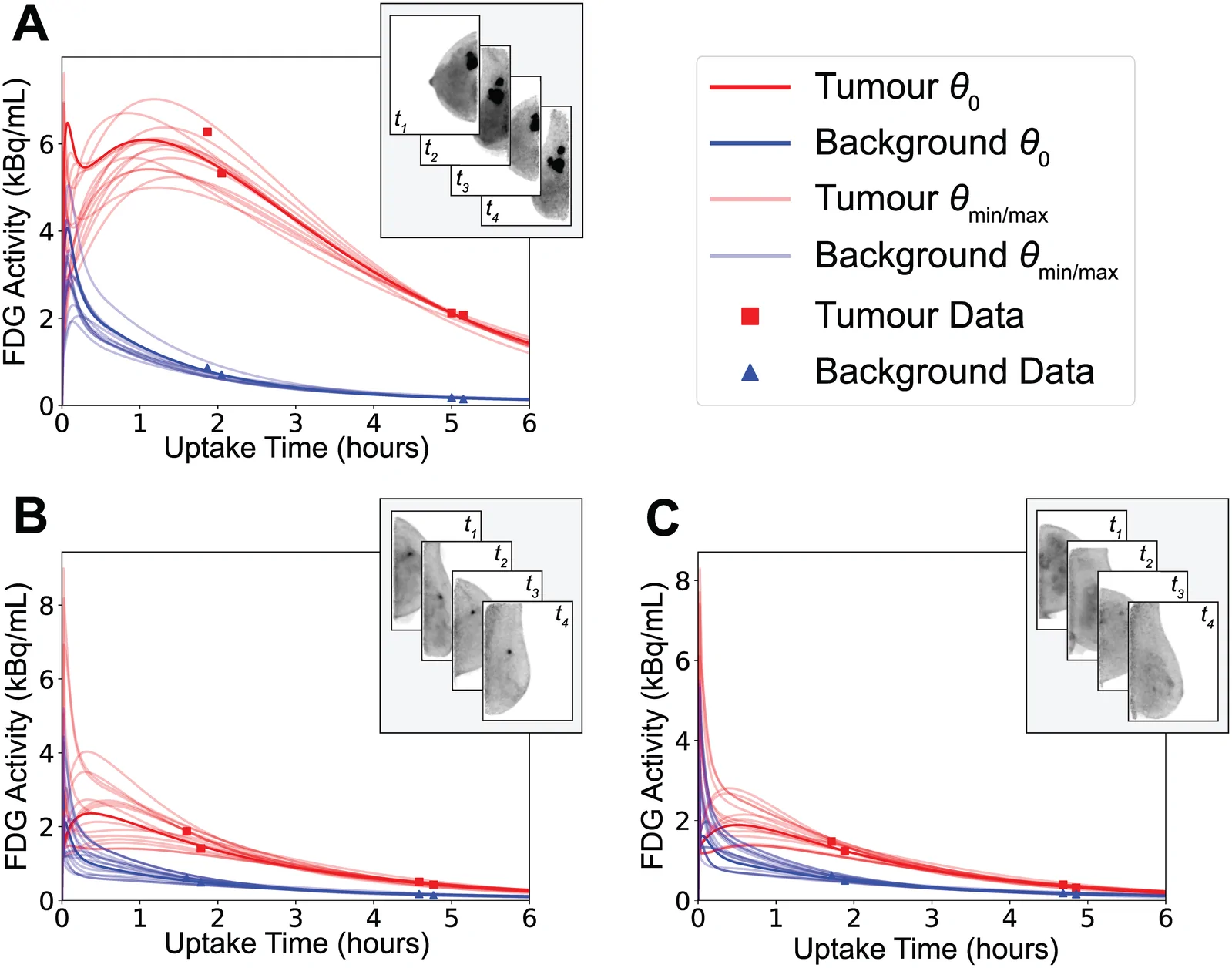
Tumour and normal (background) breast tissue time-activity curves (TACs) for three different participant image sets. Included tumours are **(A)** grade III IDC, **(B)** grade I/II IDC, and **(C)** grade III (nuclear) DCIS. The plotted points are the [^18^F]FDG activities sampled from the tumour and normal breast tissue ROIs taken from the corresponding delayed 4-image series (*t_1_, t_2_, t_3_*, and *t_4_*). Solid lines depict the TACs created with the baseline optimum (*θ*_0_) parameter sets whereas the semi-transparent lines depict the estimated range of boundary parameter sets (*θ_min/max_*).

Lower uptake tumours consequently exhibited greater tolerance for parameter variation in the *θ_min/max_* sets, as the influx and phosphorylation rate parameters (*K*_1_ and *k*_3_) in the baseline (*θ*_0_) parameter sets began further from the upper bounds. Using relative error in the optimizer objective function, *J*(*θ*), resulted in a greater absolute TAC error in regions fitted to higher activity samples. This model-fitting characteristic is visible in **Figure 5**, where tumour TAC regions around the first two samples (~2 hours) exhibit greater absolute error than those around later samples.

The estimated kinetic model parameters for the baseline (*θ*_0_) and boundary (*θ_min/max_*) sets are summarized in **Table 3**. On average, most baseline parameter sets showed a slight bias towards the minimum bounds. However, the tumour dephosphorylation rate constant (*k*_4_*_T_*) and background phosphorylation rate constant (*k*_3_*_B_*) had a notably strong bias towards the maximum and minimum bounds, respectively.

**Table 3:** Averaged baseline optimum (*θ*_0_) and boundary (*θ_min/max_*) kinetic model parameters from the 15 breast image sets compared to optimization bounds.

Parameter	*θ*_0_	*θ_min_*	*θ_max_*	Bound range
*K*_1_*_T_* (mL·g^-1^·min^-1^)	0.126	0.024	0.606	0.005–0.742
*k*_2_*_T_* (min^-1^)	0.533	0.053	2.429	0.032–2.916
*k*_3_*_T_* (min^-1^)	0.063	0.013	0.107	0.0001–0.164
*k*_4_*_T_* (min^-1^)	0.009	0.005	0.010	0.001–0.010
*K*_1_*_B_* (mL·g^-1^·min^-1^)	0.054	0.009	0.175	0.001–0.202
*k*_2_*_B_* (min^-1^)	0.327	0.053	0.778	0.008–0.902
*k*_3_*_B_* (min^-1^)	0.002	0.0007	0.011	0.0001–0.263
*k*_4_*_B_* (min^-1^)	0.004	0.001	0.008	0.001–0.010

Subscripts *T* and *B* denote the parameters belonging to the tumour and background tissue compartment models, respectively.

On average, boundary parameters utilized ~60–80% of the bounding range, except for *k*_3_*_B_*, which only utilized 4% of the bounding range. Like in the baseline parameter sets, there was a strong bias towards the lower *k*_3_*_B_* bound in the boundary parameter sets. Of note, the raw dataset used to create the background parameter bounds had an average *k*_3_*_B_* of 0.014 min^-1^. By comparison, the maximum boundary *k*_3_*_B_* had a mean of 0.011 min^-1^, indicating that the background breast tissue parameter exploration was limited to relatively mid-to-low metabolic activity.

### 3.2 Modelled CNR curves

**Figure 6** presents the modelled CNR versus uptake time curves corresponding to the same image sets used to derive the TACs in **Figure 5**. With the four CNR samples within each image set often being close in magnitude, the fitted curve regions exhibited a more balanced error distribution than the tumour TACs.

**Figure 6:**
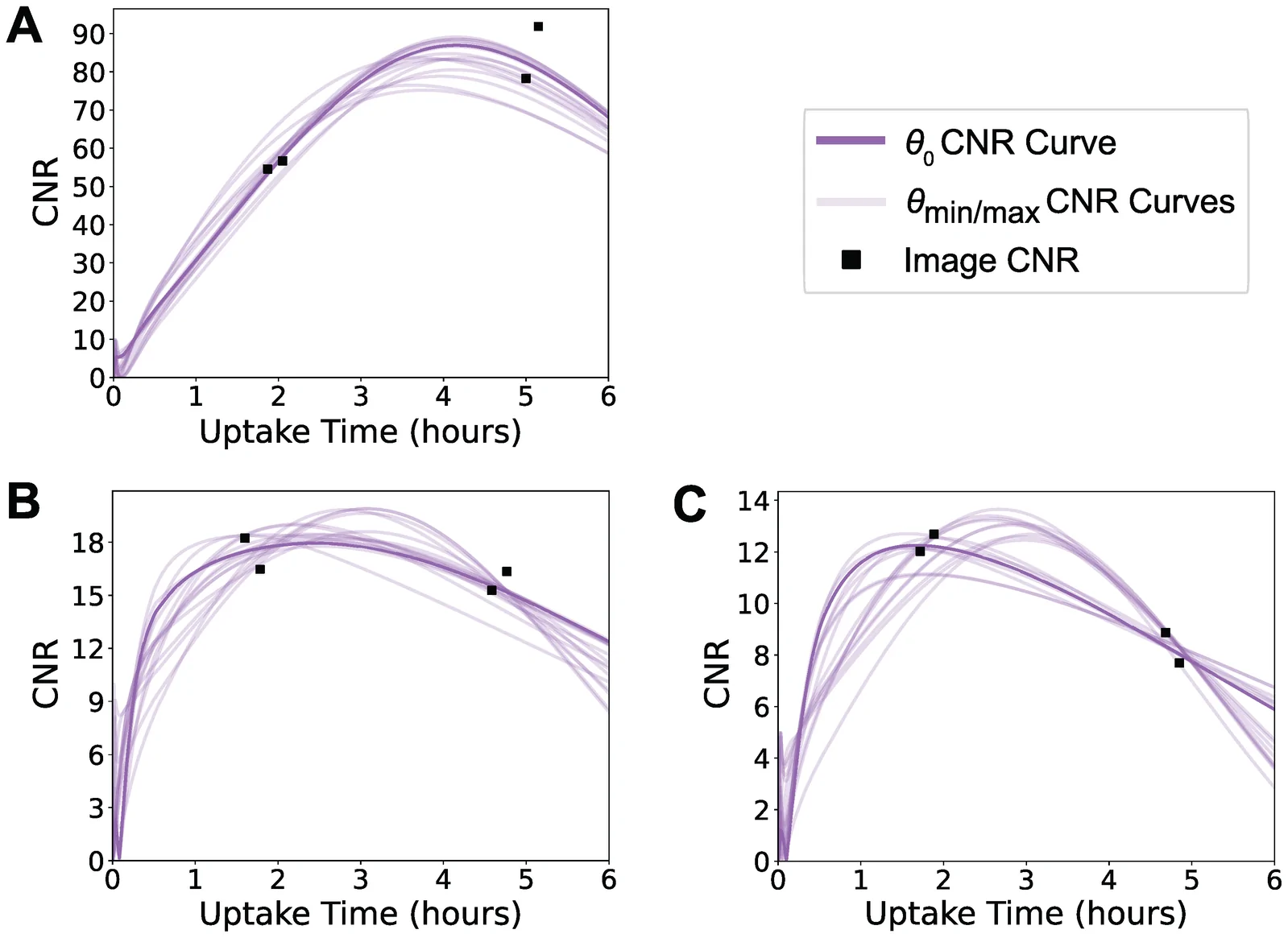
Modelled CNR vs. uptake time curves for three different participant images sets. Included tumours are **(A)** grade III IDC, **(B)** grade I/II IDC, and **(C)** grade III (nuclear) DCIS. The solid line depicts the CNR curves derived from the baseline optimized parameter set (*θ*_0_) whereas the semi-transparent lines depict the curves derived from boundary exploration parameter sets (*θ_min/max_*).

We observed that the CNR of the first two samples relative to the last two samples strongly influenced the modelled peak CNR position. For image sets in which the first two images had lower CNR than the last two, the CNR curve would typically peak around the last two images (**Figure 6A**). Conversely, when the first two images had a higher CNR than the last two, the CNR curve typically peaked around the first two images (**Figure 6C**). In cases where all four CNR samples were of very similar magnitude, the curve peak would typically be at some time between the first and last group of image CNR samples (**Figure 6B**).

### 3.3 Alternate dose simulations

To investigate the effect of different (especially lower) doses on the CNR-uptake time dependence, CNR was modelled for [^18^F]FDG doses of 1, 2, 5, and 10 mCi by adjusting the AIF scaling factor defined in Equation 2 while keeping tumour and background kinetic parameters fixed. This allowed us to examine how CNR and its peak change with uptake time at each dose level. For the representative grade III DCIS lesion in **Figure 7**, decreasing the [^18^F]FDG dose resulted in a reduction in the CNR curve magnitude. Consequently, as the dose decreased, the peak CNR decreased, and, for 75% of all parameter sets (*θ*_0_ and *θ_min/max_*), the uptake time to reach peak CNR decreased as well. This trend of later CNR peaks with increasing dose is further depicted in **Table 4**, where across the 15 lesion baseline CNR curves, the average time to peak progressively increased from 3 hours in the 1 mCi dose group, up to 3.4 hours in the 10 mCi dose group.

**Figure 7:**
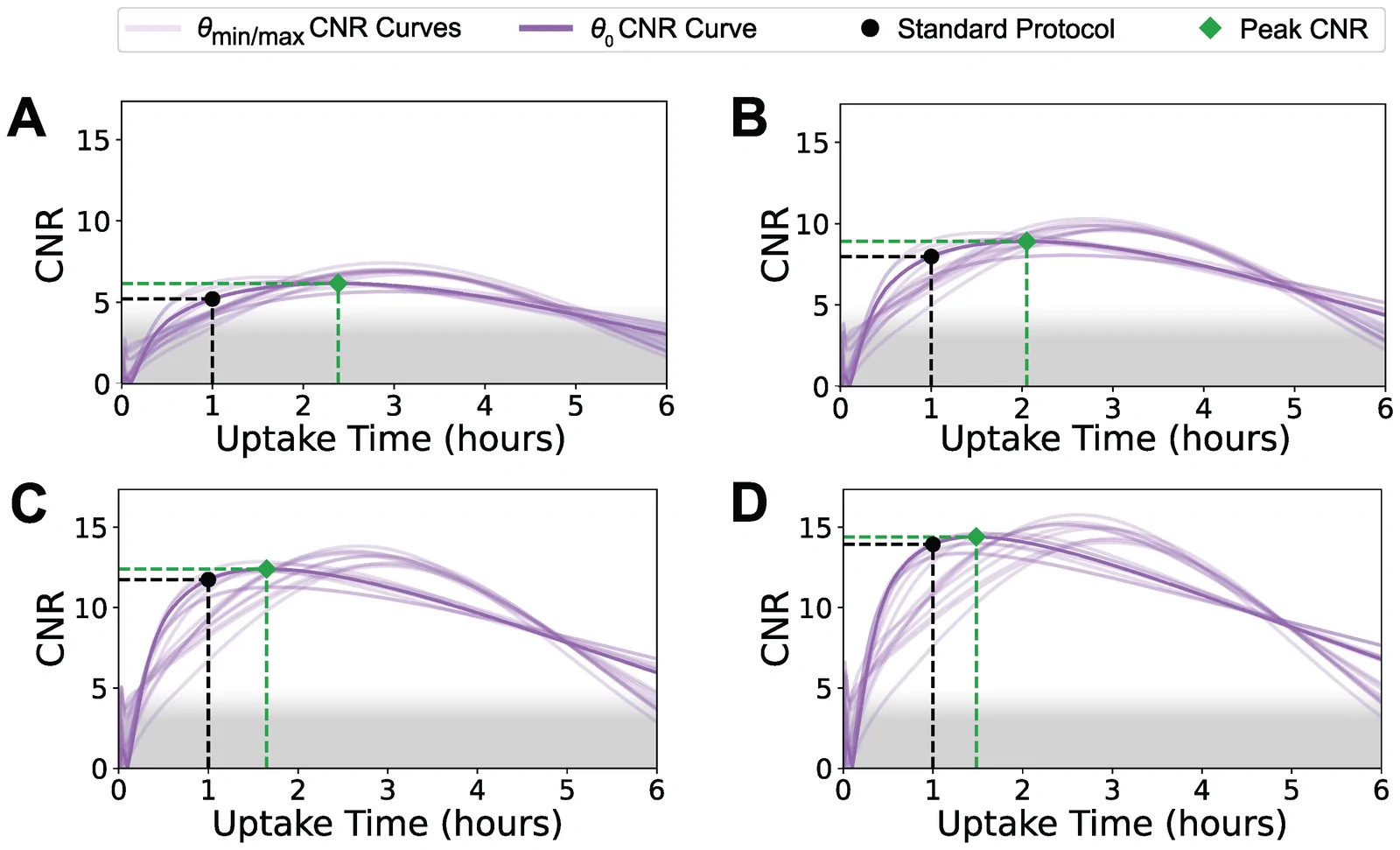
Modelled CNR vs. uptake time curves for a grade III DCIS lesion using a **(A)** 1, **(B)** 2, **(C)** 5, and **(D)** 10 mCi [^18^F]FDG dose. The solid line depicts the CNR curves derived from the baseline optimized parameter set (*θ*_0_) whereas the semi-transparent lines depict the curves derived from boundary exploration parameter sets (*θ_min/max_*). The standard protocol denoted by the black circle marker refers to the CNR at the standard 1-hour uptake time while the green diamond marker denotes the time at the peak CNR. The grey region represents where the lesion would have reduced confidence in detection (CNR< 5) per the Rose criterion.

**Table 4:** Summary of peak CNR uptake times taken from the baseline optimum (*θ*_0_) CNR curve for all lesions (*n* = 15) in each dose group.

Dose group (mCi)	Uptake time (hours)		
	Mean (SD)	Median (IQR)	Range
1	3.0 (0.8)	3.4 (1.0)	1.2–3.9
2	3.1 (0.9)	3.3 (1.2)	1.4–4.2
5	3.3 (1.1)	3.7 (1.7)	1.7–5.1
10	3.4 (1.0)	3.9 (2.0)	1.4–5.2

SD denotes standard deviation; IQR denotes interquartile range (i.e., quartile 3 - quartile 1).

### 3.4 CNR at delayed uptake times

Across all doses, CNR from the baseline optimum curves was significantly higher at every delayed uptake time than at 1 hour. The increase was approximately +30% (95% CI, 20–42%) at 1.5 hours, +53% (41–67%) at 2 hours, and +77% (62–92%) at 3 hours (Dunnett-adjusted *p* < 0.001 for all three). These estimates of percent change in CNR are reported as the linear mixed model (geometric mean) ratios, back-transformed from the log scale, with 95% confidence intervals. The estimated CNR increases were then supported by paired Wilcoxon signed-rank tests on the within-lesion log differences (Holm-adjusted within dose group), which were significant at every dose and delayed time point (all adjusted *p* < 0.001), indicating the findings did not depend on the parametric assumptions of the mixed model. CNR scaled with dose by approximately +16% (95% CI, 15–18%) per doubling of administered activity, and this dose scaling did not differ across delayed uptake times (time-by-log_2_-dose interaction *p* = 0.66).

Although 3 hours post-injection produced the largest CNR increase relative to 1 hour, it also carried the widest parameter-uncertainty envelope, and this downside was most concentrated at the 1 mCi dose. Across the full set of baseline and boundary parameter curves, the fraction with a CNR decrease at 3 hours rose as dose fell (from 6% at 10 mCi to 10% at 1 mCi), and in the worst-case at 1 mCi, the CNR decreased by 35%. At 1.5 hours, by contrast, nearly all curves remained above the 1 hour value at every dose (at most 3% showed any decrease at 1 mCi). Uptake times of 1.5–2 hours therefore produced smaller mean gains than 3 hours but still offered robust, statistically significant improvements over 1 hour with markedly less downside variability at the lowest doses.

### 3.5 Assessment of lesion uptake

The effects of uptake time and dose on tumour TACs were analysed using the SUV_peak_. More specifically, we analysed the magnitude of change in SUV_peak_ at 10-minute intervals from 1 hour up to 1.5 hours post-injection. Notably, at 1 hour versus 1.5 hours, the SUV_peak_ changed on average by 14% in the 1 mCi group and progressively increased up to 17% in the 10 mCi group. In each dose group, at 1 hour versus 1.5 hours, the SUV_peak_ changed by up to 30% for some TACs. Within the first 10-minute interval (from 1 hour), the SUV_peak_ increased on average by 5–6% across dose groups, reaching as high as 11% for some TACs.

### 3.6 Assessment of detectability

The overall confidence in tumour detectability (assessed using the Rose criterion for confident detection with CNR ≥ 5) increased with [^18^F]FDG dose. Regardless of dose, all invasive lesions (i.e., non-DCIS) achieved CNR ≥ 5 by 1 hour post-injection and remained above this level across all simulated doses (1, 2, 5, and 10 mCi).

Sub-threshold detectability at 1 hour was confined to a single DCIS lesion (grade III). On its baseline (*θ*_0_) curve, this lesion exceeded CNR ≥ 5 at 1 hour even at 1 mCi (CNR = 5.2); however, its detectability at the 1 mCi dose was sensitive to kinetic-parameter uncertainty. Across the 16 boundary (*θ_min/max_*) CNR curves spanning the parameter-uncertainty envelope, only 4 exceeded CNR ≥ 5 at 1 hour in the 1 mCi group (**Figure 7A**). By 1.5 hours post-injection, 12 of 16 boundary curves surpassed the threshold, and by 2 hours all curves indicated confident detectability (CNR ≥ 5). At 2 mCi, the same lesion had 15 of 16 boundary curves above threshold at 1 hour, with all 16 confidently detectable by 1.5 hours; at 5 and 10 mCi it was detectable across the full envelope at all uptake times.

## 4 Discussion

The primary aim of this study was to provide a systematic framework for optimizing low-dose, [^18^F]FDG PEM protocols by simulating a range of dose and uptake time combinations. Conventional PET/CT imaging strategies are designed around injected doses of 10 mCi or higher, with a 1-hour uptake time that may be unfavourable for low-dose PEM. Our central finding is that extending the uptake time beyond the standard 1-hour clinical protocol may significantly improve CNR across low to standard doses, with a practical benefit observed between 1.5 and 2 hours post-injection. Notably, even at a dose of 1 mCi, a modest 30-minute increase from the 1-hour standard yielded an approximately 30% increase to the CNR. These results indicate that PEM protocol design may benefit from accounting for dose and system differences, offering preliminary quantitative support for adapted imaging strategies rather than a uniform approach.

Our simulations demonstrated that uptake times beyond the 1-hour clinical standard significantly improved CNR across all assessed [^18^F]FDG dose groups, increasing by approximately 30%, 53%, and 77% at 1.5, 2, and 3 hours relative to the 1-hour standard, with comparable proportional gains at every dose. Peak CNR tended to occur around 3–3.4 hours post-injection; however, such prolonged uptake times risk disrupting scheduling in routine clinical workflows and, at the lowest dose (1 mCi), were associated with a wider uncertainty envelope in the modelled CNR. Uptake times of 1.5–2 hour provided substantial CNR improvements over the 1-hour standard while remaining more operationally practical and avoiding the less predictable behaviour observed at 3 hours under low-dose conditions. The clinical relevance of this moderate extension is illustrated by a low-grade DCIS lesion in our cohort: at 1 mCi and 1 hour post-injection, only 4 of 16 modelled boundary CNR curves met the Rose criterion (CNR ≥ 5); by 1.5 hours, 12 of 16 curves met this threshold; and by 2 hours, all 16 did so. This finding demonstrates that within the uncertainty envelope for this lesion, a modest acquisition delay may convert a borderline, sub-threshold lesion into one that is confidently detectable under low-dose conditions.

This finding is particularly significant when considering low administered doses, such as 1 mCi, since the average effective whole-body dose falls below 1 mSv and is comparable to that of digital mammography. More specifically, the observed effects of delayed imaging at such low doses could be of benefit to PEM-based screening in dose-sensitive populations, including women undergoing routine high-risk surveillance (11, 14). Our simulations therefore support a low-dose imaging strategy in which modestly extended uptake times of approximately 1.5–2 hours enhance lesion conspicuity at 1–2 mCi while maintaining radiation exposure within mammography-equivalent ranges. Importantly, this study is among the first to demonstrate that breast PET image quality gains associated with delayed imaging are achievable at low doses; prior literature had investigated this relationship exclusively at standard-to-high doses (i.e., 6–20 mCi) (19–22). The present findings suggest that the biological mechanisms underlying improved CNR at delayed imaging time can still be leveraged in low-activity protocols.

The existing literature at standard and high [^18^F]FDG doses points to two complementary mechanisms that explain improved breast tumour contrast at delayed uptake times: progressive washout of background breast tissue activity and a peak detectability window before radioactive decay noise becomes dominant. Kim et al. demonstrated that at 2 hours post-injection, washout of background activity led to increased tumour contrast compared to 1 hour, with the effect most pronounced in participants with higher background uptake (22). Similarly, Baun et al. reported that decreased background uptake over time led to an increased tumour-to-background ratio at 3 hours versus 1 hour (21). Wangerin et al. found that nearly half of their participants (11 of 23) reached peak detectability at 2–3 hours post-injection using an irreversible kinetic model (20), while Boerner et al. reported that tumour-to-background ratios were significantly higher at 3 hours compared to 1.5 hours, with lesion detectability improving from 83% to 93% (19). Taken together, these studies consistently affirm the benefit of delayed breast PET imaging observed in the present study.

The prior studies cited above employed doses of 6 mCi or higher and reported peak contrast at approximately 2–3 hours post-injection, which is broadly consistent with our findings in the 5 and 10 mCi dose groups, where peak CNR occurred at approximately 3.3 to 3.4 hours on average, and up to 5 hours in some cases. The slightly later peak observed in our higher-dose groups may reflect the superior count sensitivity and spatial resolution of the Radialis system, which likely extends the window of favourable imaging characteristics beyond 3 hours relative to the WB PET/CT scanners used in the comparative studies. It is also worth noting that only one of the cited studies imaged beyond 3 hours (20), limiting direct comparison at later time points.

A secondary finding of this study is the significant and rapid changes in the standardized uptake value (SUV) from 1 hour post-injection onward, driven by ongoing tumour uptake. Even within a 10-minute window starting at 1 hour post-injection, SUV_peak_ changed by an average of 5–6%, with individual instances reaching as high as 11%. These rapid temporal changes in tumour activity have critical implications for any application requiring precise quantification of [^18^F]FDG uptake, most notably for early tumour response assessment during neoadjuvant systemic treatment (NST) for breast cancer, where prompt identification of non-responders can guide transitions to more effective regimens (37). While the European Association of Nuclear Medicine currently accepts follow-up imaging within ±10 minutes of the initial uptake time (38), our results suggest that even such small timing discrepancies may meaningfully affect the confidence and accuracy of metabolic activity quantification.

Certain limitations constrained our proposed modelling method: First, we sampled uptake data from four delayed static PEM images per lesion, rather than a dynamic imaging acquisition done over the first hour post-injection. However, by jointly fitting tumour and background TACs, and leveraging published kinetic parameter data for normal and malignant breast tissue to derive physiological bounds (20, 35), we were able to narrow the parameter estimation search space despite limited image samples. Second, the arterial input functions (AIFs) used in the modelling were population-based rather than individualized, which reduces accuracy relative to patient-specific methods derived from serial blood sampling or image-derived input functions (34). Nevertheless, this approach demonstrates utility in using population-based parameters to infer high-level CNR trends with respect to uptake time; integrating dynamic imaging and patient-specific AIFs in future studies could further strengthen the methodology.

As this study was exploratory and derived from a small pilot evaluation (n = 13 participants, 15 lesions), these findings are hypothesis-generating rather than confirmatory, and the reported *p*-values should be interpreted with corresponding caution. Of the lesions sampled for modelling, all were ≥1.2 cm and drawn from symptomatic patients undergoing diagnostic work-up, so the generalizability to subcentimeter lesions and an asymptomatic screening population remains unclear. While CNR is an appropriate and widely used characteristic of PET image quality, it does not directly quantify changes in diagnostic performance (25). Thus, the estimated CNR gains and application of the Rose criterion should be interpreted as a quantitative measure of image quality that facilitates but does not necessarily lead to improved diagnostic sensitivity. Lastly, the quantitative results are specific to the Radialis PET Imager’s detector performance characteristics, though the underlying background-washout mechanism should generalize to other high-sensitivity PEM systems. Future large-scale clinical trials using high-sensitivity PEM systems such as the Radialis PET Imager will be essential to validate these simulation-based findings in prospective patient cohorts.

## 5 Conclusion

In this study, we used kinetic modelling of tumour and background time-activity curves to provide a quantitative rationale for adjusting [^18^F]FDG uptake times in low-dose PEM protocols. Our simulations indicate that, when injected activity is reduced to approximately 1 and 2 mCi—an activity range that is particularly relevant for dose-sensitive applications such as potential future PET-based breast screening with high-sensitivity PEM—lesion conspicuity may be improved by extending the acquisition window to 1.5–2 hours post-injection. These findings warrant further investigation into refining low-dose [^18^F]FDG PEM protocols to facilitate clinical implementation of high-sensitivity breast imaging for large cohorts of women in whom conventional X-ray mammography is inefficient, including those with dense breasts or at elevated risk of breast cancer. We also demonstrate that modest variations in uptake time can substantially affect SUV, underscoring the need for tighter standardization of uptake time in follow-up imaging protocols used for treatment-response assessment.

## Data Availability

The raw data supporting the conclusions of this article will be made available by the authors, without undue reservation.
