# Supplementary material for: Modelling image quality over extended [18F]FDG uptake times for low-dose positron emission mammography
Source: Front Oncol. 2026 Sep 14;16:1880803. doi: 10.3389/fonc.2026.1880803 (PMC13616630; doi:10.3389/fonc.2026.1880803)
Supplement: Supplementary file 1 [file Supplementaryfile1.docx]

Supplementary Material

# 1 Supplementary Methods

## 1.1 Assessment of normality and choice of analysis scale

We assessed the normality of the CNR change from 1 hour post-injection to the delayed time points (1.5, 2, and 3 hours) using Shapiro–Wilk tests in R (v4.6.1; R Core Team 2026) on the paired CNR differences from the baseline curves (n = 15 lesions), across all 12 dose–time-point combinations (4 dose groups × 3 time points), summarized in Supplementary Table 1. From the Shapiro–Wilk test, it became clear that the CNR change was right-skewed and non-normal at every combination (Shapiro–Wilk *p* < 0.05 for all 12). We repeated this test using the log CNR ratios (Supplementary Table 2), which, conversely, showed no evidence against normality (all *p* > 0.15), supporting the use of the log scale in further analysis.

## 1.2 Primary test and robustness checks

The primary analysis is a linear mixed-effects model of log CNR (fitted with the lme4 and lmerTest R packages), with time point and dose as fixed effects and random intercepts for lesion nested within participant. Since we found the time-by-dose interaction was negligible (see Supplementary Results, the primary model pooled the time effect across dose. Pre-planned contrasts compared each delayed time point (1.5, 2, and 3 hours) with the 1 hour reference, with Dunnett-style adjustment for multiplicity (emmeans R package). These choices were supported by the distributional diagnostics in the previous Shapiro-Wilk test results and by approximately normal model residuals on a quantile–quantile plot (Supplementary Figure 1). To confirm the conclusions did not depend on parametric assumptions, all comparisons were additionally evaluated with paired Wilcoxon signed-rank tests on the log scale (Holm-adjusted within dose), which were concordant with the mixed model (Supplementary Table 3).

# 2 Supplementary Results

Across all doses, CNR increased significantly relative to the 1-hour reference at every delayed time point: by approximately +30% (95% CI, 20–42%) at 1.5 hours, +53% (41–67%) at 2 hours, and +77% (62–92%) at 3 hours (Dunnett-adjusted *p* < 0.001 for all three). These estimates are reported as the linear mixed model (geometric mean) ratios, back-transformed from the log scale, with 95% confidence intervals. The time-by-dose interaction was negligible (F(9, 210) = 0.19, *p* = 0.995), indicating that the proportional CNR gain from delayed imaging did not differ across the four simulated doses; the primary model therefore pooled the time effect across dose. Per-dose contrasts are given in Supplementary Table 4.

CNR scaled with administered activity by approximately +16% per doubling of dose and this dose scaling did not differ across time points (time-by-log₂-dose interaction *p* = 0.66). Re-fitting the model on each lesion’s median across all 17 curves reproduced the estimates, and the log-scale Wilcoxon tests were concordant with the mixed model (Supplementary Table 3). Under the Rose criterion (CNR ≥ 5), all 15 lesions were detectable at every dose and time point on the baseline curve; across the full uncertainty envelope ($\theta_{min/max}$), detectability was universal except for one lesion at 1 mCi at the 1-hour and 1.5-hour time points, whose plausible range straddled the threshold and resolved by 2 hours.

# 3 Supplementary Figures and Tables

## 3.1 Supplementary Figures

**Supplementary Figure 1.** Quantile–quantile plot depicting the normality of the linear mixed model. The plotted points (n = 240) arise from each CNR observation where there are 15 lesions × 4 doses × 4 post-injection time points (1, 1.5, 2, 3 hours).

## 3.2 Supplementary Tables

**Supplementary Table 1.** Shapiro-Wilk test results for the CNR difference relative to 1 hour post-injection at all dose-time-point combinations, using the baseline parameter curves (n = 15 lesions).

| Dose Group (mCi) | Timepoint (hours) | *p* | Skew |
| --- | --- | --- | --- |
| 1 | 1.5 | < 0.05 | 1.39 |
| 1 | 2 | < 0.05 | 1.22 |
| 1 | 3 | < 0.05 | 0.86 |
| 2 | 1.5 | < 0.05 | 1.58 |
| 2 | 2 | < 0.05 | 1.45 |
| 2 | 3 | < 0.05 | 1.14 |
| 5 | 1.5 | < 0.05 | 1.61 |
| 5 | 2 | < 0.05 | 1.55 |
| 5 | 3 | < 0.05 | 1.40 |
| 10 | 1.5 | < 0.05 | 1.55 |
| 10 | 2 | < 0.05 | 1.52 |
| 10 | 3 | < 0.05 | 1.45 |

Note: *p*-values < 0.05 for the Shapiro-Wilk test indicate significant departure from normality.

**Supplementary Table 2.** Shapiro-Wilk test results for the log ratio of CNR at a given time point relative to 1 hour post-injection at all dose–time-point combinations, using the baseline parameter curves (n = 15 lesions).

| Dose Group (mCi) | Timepoint (hours) | *p* | Skew |
| --- | --- | --- | --- |
| 1 | 1.5 | 0.578 | -0.34 |
| 1 | 2 | 0.485 | -0.41 |
| 1 | 3 | 0.374 | -0.51 |
| 2 | 1.5 | 0.328 | -0.20 |
| 2 | 2 | 0.315 | -0.23 |
| 2 | 3 | 0.316 | -0.24 |
| 5 | 1.5 | 0.167 | -0.15 |
| 5 | 2 | 0.187 | -0.15 |
| 5 | 3 | 0.156 | -0.09 |
| 10 | 1.5 | 0.288 | -0.16 |
| 10 | 2 | 0.316 | -0.15 |
| 10 | 3 | 0.251 | -0.07 |

**Supplementary Table 3.** Paired Wilcoxon signed-rank test results on the log ratio of CNR at each time point relative to 1 hour post-injection, at all dose-time-point combinations, using the baseline parameter curves (n = 15 lesions).

| Dose Group (mCi) | Timepoint (hours) | *p* |
| --- | --- | --- |
| 1 | 1.5 | < 0.001 |
| 1 | 2 | < 0.001 |
| 1 | 3 | < 0.001 |
| 2 | 1.5 | < 0.001 |
| 2 | 2 | < 0.001 |
| 2 | 3 | < 0.001 |
| 5 | 1.5 | < 0.001 |
| 5 | 2 | < 0.001 |
| 5 | 3 | < 0.001 |
| 10 | 1.5 | < 0.001 |
| 10 | 2 | < 0.001 |
| 10 | 3 | < 0.001 |

Note: *p*-values are Holm-adjusted within dose.

**Supplementary Table 4.** Per-dose CNR increase relative to 1 hour post-injection (linear mixed-effects model on log CNR; back-transformed to percent change).

| Dose (mCi) | Timepoint (hours) | % change vs 1 h | 95% CI | *p* |
| --- | --- | --- | --- | --- |
| 1 | 1.5 | +29.2 | 8.2–54.3 | 0.002 |
| 1 | 2 | +49.8 | 25.5–78.9 | < 0.001 |
| 1 | 3 | +65.8 | 38.9–98.0 | < 0.001 |
| 2 | 1.5 | +30.1 | 9.0–55.4 | 0.002 |
| 2 | 2 | +52.4 | 27.6–81.9 | < 0.001 |
| 2 | 3 | +73.3 | 45.1–106.9 | < 0.001 |
| 5 | 1.5 | +30.9 | 9.6– 56.2 | 0.001 |
| 5 | 2 | +54.7 | 29.6– 84.7 | < 0.001 |
| 5 | 3 | +81.7 | 52.2–117.0 | < 0.001 |
| 10 | 1.5 | +31.1 | 9.8–56.5 | 0.001 |
| 10 | 2 | +55.7 | 30.4– 85.9 | < 0.001 |
| 10 | 3 | +86.0 | 55.8–122.1 | < 0.001 |

Note: 95% CI denotes 95% confidence intervals for % change in CNR vs 1 hour post-injection; *p*-values are Dunnett-adjusted within dose.
